# Oral Management by a Full-Time Resident Dentist in the Hospital Ward Reduces the Incidence of Pneumonia in Patients with Acute Stroke

**DOI:** 10.1155/2022/6193818

**Published:** 2022-07-22

**Authors:** Kenichiro Ozaki, Satoshi Teranaka, Haruka Tohara, Shunsuke Minakuchi, Satoru Komatsumoto

**Affiliations:** ^1^Department of Physical Medicine and Rehabilitation, Japanese Red Cross Ashikaga Hospital, Ashikaga, Japan; ^2^Dysphagia Rehabilitation, Graduate School of Medical and Dental Sciences, Tokyo Medical and Dental University, Tokyo, Japan; ^3^Gerodontology and Oral Rehabilitation, Graduate School of Medical and Dental Sciences, Tokyo Medical and Dental University, Tokyo, Japan; ^4^Department of Internal Medicine, Japanese Red Cross Ashikaga Hospital, Ashikaga, Japan

## Abstract

**Background:**

A full-time dentist was assigned to a ward at our hospital to improve the quality of oral healthcare for hospitalized patients. A dental care system (DCS) was created to facilitate the collaboration between the full-time dentist and the nursing department.

**Objective:**

To investigate the effects of DCS implementation on the incidence of pneumonia in patients with acute stroke.

**Methods:**

This retrospective cohort study comprised 945 hospitalized acute stroke patients categorized into three groups: pre-, during-, and post-DCS. The DCS comprised dentist-led lectures and practical sessions, oral assessments, standardized oral care techniques, and information on the procedures for nurse-requested dental intervention. Data were extracted from the Japanese Diagnosis Procedure Combination database and medical records. The attributes of the patients, incidence of pneumonia, and number of patients who requested dental intervention were determined.

**Results:**

The odds ratios of pneumonia onset were 3.16 (95% confidence interval [CI], 1.65–6.05; *P* = 0.001) in the pre-DCS and 2.80 (95% CI, 1.48–5.31; *P* = 0.002) in the during-DCS group compared with the post-DCS group, thereby confirming the effect of DCS on the incidence of pneumonia. The number of dental requests in the post-DCS group was noted to be higher than that in the pre-DCS group (*P* = 0.002).

**Conclusion:**

Oral management by a full-time dentist was found to be effective in reducing the incidence of pneumonia in patients with acute stroke. To implement the best oral care practices in the hospital wards, the full-time dentist should work as a member of the medical team.

## 1. Introduction

Approximately 1 in every 10 stroke patients is reported to experience pneumonia during hospital care [1]. Furthermore, poor oral hygiene and missing teeth not only increase the incidence of pneumonia [2] but also have a negative effect on the outcome of rehabilitation after stroke [3]. According to the guidelines for stroke patients [4], an early dental checkup is recommended for patients with poor oral hygiene; systematic oral care has been shown to reduce the incidence of pneumonia [5]. However, the availability of full-time dentists assigned to provide interventions to patients with systemic conditions during their stay at the hospital remains limited.

In our hospital, full-time dentists specializing in inpatients belong to the department of rehabilitation, which enables them to manage the oral conditions of the patients in a timely manner. In addition, house calls are made to the wards when dealing with acute patients, thus facilitating cooperation with the ward nurses. Oral care in the elderly is of low priority among nurses in acute hospitals [6]. In such instances, oral management by dentists during the acute stage might prove beneficial for these patients. Accordingly, we have established a dental care system (DCS) in our hospital, in which full-time dentists who work exclusively in the wards collaborate with the nursing department to manage the oral conditions of the patients. In this study, we aimed to evaluate the effect of the DCS on the development of pneumonia in acute stroke patients.

## 2. Methods

### 2.1. Study Population and Design

**Figure 1:**
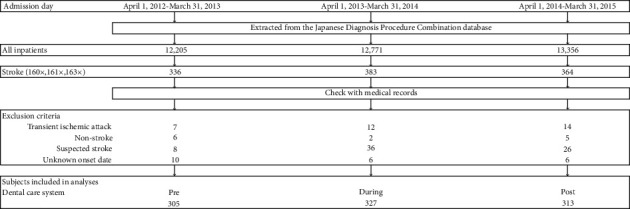
Flowchart of patient recruitment.

This is a retrospective cohort study comprising stroke patients who were admitted to the Japanese Red Cross Ashikaga Hospital, which is a tertiary emergency facility accredited by the Joint Commission International, between April 1, 2012, and March 31, 2015. The patients were selected from the Japanese Diagnosis Procedure Combination database [7] based on the diagnosis of cerebral infarction (I63x), cerebral hemorrhage (I61x), or subarachnoid hemorrhage (I60x) at the time of admission. The cases were reviewed from the medical records (Hope Egmain-Gx, Fujitsu, Tokyo, Japan); patients with transit ischemic attack, suspected stroke, and those with unknown onset dates were excluded from analysis. Subsequently, patients with cerebral infarction (International Classification of Diseases, 10th [ICD-10], code I63x; WHO 2010), cerebral hemorrhage (ICD-10, code I61x; WHO 2010), and non-traumatic subarachnoid hemorrhage (ICD-10, code I60x; WHO 2010) were identified and categorized into the three phases of the DCS: pre-introduction, transition, and establishment phases (Figure 1). Pre-introduction was defined as the period during which the incorporation of DCS into nursing care was being considered (Pre-DCS; April 1, 2012 to March 31, 2013). Transition was defined as the period during which we provided education to establish DCS (During-DCS; April 1, 2013 to March 31, 2014). Establishment was defined as the period when DCS practice had come into widespread use (Post-DCS; April 1, 2014 to March 31, 2015).

This study was reviewed and approved by the Ethics Committee of Japanese Red Cross Ashikaga Hospital (authorization ID 2020–12). Informed consent was obtained from all patients or their agents upon admission.

### 2.2. Data Collection

The following information was obtained from the medical records at the time of admission: classification of stroke, admission by ambulance, admission from home, sex, age, body mass index (BMI; <18.5 kg/m^2^), Japan Coma Scale (JCS; 0, 1, 2, and 3 digits), and the presence of comorbidities (stroke, dementia, heart failure, renal failure, hypertension, respiratory disease, hyperlipidemia, diabetes, and liver disease). Additionally, information about the interventions provided within 3 days of hospitalization (surgery, speech-language pathologist [swallowing rehabilitation], and dentistry), the occurrence of pneumonia, and the duration of hospital stay were collected. The JCS has been widely used in Japan to assess the level of consciousness and is reported to correlate with the Glasgow Coma Scale [8]. The JCS scores are divided into four main categories as follows: 0 indicates alert consciousness, single-digit scores signify that the patient is awake without any stimuli, double-digit scores denote patients who can be aroused with stimuli, and triple-digit scores indicate coma. A diagnosis of pneumonia was made by the attending physician according to the CDC/NHSN surveillance definition of the healthcare-associated infection and the specific type of infection: pneumonia (PNU1) [9]. The onset of pneumonia within 2 days of hospitalization was excluded because it does not conform to the definition of hospital-acquired pneumonia [10]. The number of patients, number of visits, number of days between admission and intervention, nature of the intervention, number of the remaining teeth at the first visit, Eichner's classification [11], denture ownership, crusting on the lips, xerostomia, and dysphagia were surveyed among the patients who underwent dental interventions. Dysphagia is a condition characterized by the difficulty in swallowing saliva, thereby requiring pharyngeal suction. Patients whose dentures had been adjusted were checked for denture use at discharge. Dental intervention patients were identified as those who were examined by a dentist at the first visit and had received interventions by a team of dental hygienists.

### 2.3. The DCS

The department of rehabilitation medicine at our hospital comprises a multidisciplinary team, including two full-time dentists and two dental hygienists specialized in geriatric dentistry, particularly, inpatients. The DCS created for nurses consists of lectures and practical training that are conducted by a dentist and a dental hygienist, oral assessments, standardized oral care procedures, and procedures for requesting dental intervention from the nurses. The nurses are required to attend a 1-hour lecture and a 1-hour practical session conducted by a dentist and a dental hygienist. The lectures cover various topics such as the effects of oral care, oral anatomy, saliva, oral care products, oral assessment, risk assessment, and oral care procedures. A self-made oral care video is used for practical training. After watching the video, the nurses are required to undergo an interactive practical session using mucous membrane brushes, toothbrushes, and oral moisturizers. The assessment part involves evaluations for the presence of phlegm, dryness, food residue, tongue coating, ulcers, and bleeding. Each item consists of a “yes/no” option, with a column for additional comments (Supplementary Table 1). The evaluations are conducted by the nurses every day from the time of admission with the aid of a penlight. Patients who can independently perform oral care are assessed after brushing following meals; patients requiring assistance are assessed by the nurses before oral care. Assessments are terminated when all items are negative for three consecutive days, but the patients are reassessed after 1 week or when their condition worsens. For the standardization of the oral care procedures, the nurses are required to learn the following oral care techniques to tend to patients who require assistance: (1) apply an oral moisturizer or white petroleum jelly if there is crusting on the lips; (2) apply an oral moisturizer using an oral mucosa brush if there is dry sputum or exfoliated epithelium in the mouth; (3) remove contaminants using an oral mucosa brush in the following order: lip and cheek mucosa, gingiva, palate, tongue, and floor of the mouth; (4) brush the natural teeth; (5) wipe the residual contaminants using a brush for the oral mucosa and apply an oral moisturizer if the dryness is severe; and (6) remove the denture plaque if dentures are present. The information is saved in a shared file in the electronic medical record and made available for re-learning. In addition, illustrated materials related to oral care procedures and products have been laminated and presented as oral care charts in the wards. Oral care is performed after each meal for patients who consume foods orally and require assistance. Oral care is performed based on the oral contamination pattern classification (Supplementary Table 1), which is defined as follows: A, dry sputum (+) and pharyngeal suction (+); B, viscous sputum (+) and pharyngeal suction (+); C, xerostomia (+) and pharyngeal suction (−); and D, xerostomia (−) and pharyngeal suction (−). The frequency of oral care for parenteral patients who require assistance is defined as two to three times a day for pattern A, twice a day for pattern B, and one to two times a day for patterns C and D. The nurses can request dental care if any assessment item is positive for more than 3 days; if toothache, tooth mobility, ulcers, bleeding, denture incompatibility, or difficulty in opening the mouth is observed; and if the oral care is judged to be difficult. To establish the DCS, a committee for oral care was established by dentists and nurses during the transition period, and monthly meetings were held to reconfirm the oral assessment and healthcare techniques and to survey the use of oral care products. One representative nurse from each ward participated in the committee.

### 2.4. Dental Procedures

All dental interventions were performed in the patient's room at the hospital. When drilling the teeth or denture adjustment was necessary, a portable dental unit or handpiece was used depending on the stroke pathology. The primary oral care products included a tongue brush, sponge brush for oral care, oral moisturizer, and white petroleum jelly. Patients with teeth were given a toothbrush, an antiseptic mouthwash containing benzethonium chloride (NEOSTELIN ®GREEN 0.2% Mouthwash Solution, Nippon Shika Yakuhin Co. Yamaguchi, Japan), an interdental brush, and a tufted brush. Finger guards were used when there was difficulty in opening the mouth. Petroleum jelly was prescribed in cases of lip dryness or crusting. For stomatitis, dexamethasone (Dexaltin® oral ointment, 1 mg/g; Nippon Kayaku Co., Tokyo, Japan) was prescribed. If positive for oral candida, drugs for oral and esophageal candidiasis (FLORID® oral gel 2%, Mochida Pharmaceutical Co., Tokyo, Japan) were used. For bleeding, pressure was applied with gauze, and if this failed to achieve hemostasis, adrenaline solution (BOSMIN® Solution, Daiichi Sankyo Co., Tokyo, Japan) was used. Tooth extraction was performed with a local anesthetic (ORA®Inj. Dental Cartridge 1.8 ml, Showa Yakuhin Kako Co., Tokyo, Japan) for teeth with grade 3 mobility (Miller's mobility index) [12]. Denture adjustment was performed at the start of oral intake of meals. Moreover, dental interventions were performed in the pre-DCS group.

### 2.5. Sample Size Calculation

The sample size was calculated based on a previously published study in which the incidence of pneumonia was 7% in the intervention group and 16% in the control group [13]. When this was set to a power of 0.80 and a significance level of 0.05, the sample size was 196 in each group (online sample size calculator, ClinCalc).

### 2.6. Statistics

Comparisons were made among the three groups: pre-, during-, and post-DCS. In the univariate analysis, stroke classification, admission by ambulance, admission from home, sex, age, BMI at admission, JCS at admission, presence of comorbidities, interventions within 3 days of hospitalization (surgery, speech-language pathologist, dentistry), the incidence of pneumonia after 3 days of hospitalization, number of dental patients, contents of dental treatment, number of remaining teeth, Eichner's classification, denture ownership, crusting on the lips, xerostomia, and dysphagia were analyzed using Pearson's chi-square test. The length of hospital stay, number of dental visits, and number of days between admission and dental intervention were determined via the Wilcoxon's signed-rank test. Multiple comparison tests were performed using Bonferroni's correction test. Multivariate analysis was performed to evaluate the association between the DCS and pneumonia. The onset of pneumonia was used as the objective variable, and sex, BMI (less than 18.5 vs. 18.5 or more), JCS (2 and 3 digits vs. 0 and 1 digit), heart failure, respiratory disease, intervention by a speech-language pathologist within 3 days of hospitalization, and DCS (pre, during vs. post; pre vs. during) were used as the explanatory variables. The discrimination was measured using the area under the receiver operating characteristic curve (AUC).

The statistical software JMP14.3 for Macintosh (SAS Institute Inc., Cary, NC, USA) was used for statistical analyses, and a *P*-value of <0.05 was considered significant.

## 3. Results

**Figure 2:**
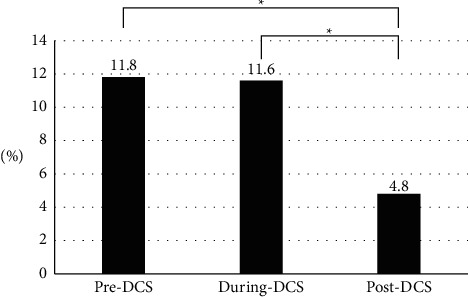
Rate of pneumonia onset for the three DCS (dental care system) time periods studied. ^*∗*^*P* < 0.05 chi-square test, Pre-DCS versus Post-DCS, During-DCS versus Post-DCS All comparisons used the Pearson's chi-square test, and multiple comparisons used Bonferroni's adjustment.

The total number of patients included in this study was 945 (females, 46%; mean age, 72 ± 13 years); among them, 305 patients (females, 50%; mean age, 72 ± 13 years) were categorized into the pre-DCS group, 327 (females, 42%; mean age, 71 ± 13 years) into the during-DCS group, and 313 (females, 45%; mean age, 73 ± 12 years) into the post-DCS group. The mean duration of hospital stay for pneumonia follow-up in the three groups were as follows: 28 ± 24 days in the pre-DCS group, 28 ± 27 days in the during-DCS group, and 30 ± 33 days in the post-DCS group. The demographic characteristics were not significantly different between the three groups.  Table 1 shows the results of the univariate analysis of the characteristics of the patients at admission and the intervention status (within 3 days of admission). The number of dental interventions within 3 days of admission was significantly higher in the post-DCS group when compared to those in the pre- and during-DCS groups (*P* < 0.001). No significant differences were observed with the other items. The incidence of pneumonia in the post-DCS group (4.8%) was noted to be significantly lower than those in the pre-DCS (11.8%) and during-DCS (11.6%) groups (*P* < 0.017; Figure 2). The results of the multivariate analysis are shown in Table 2. The odds ratios (95% confidence interval [CI]) of pneumonia after 3 days of hospitalization were 3.16 (95% CI, 1.65–6.05; *P*=0.012; *P*=0.001) in the pre-DCS and 2.79 (95% CI, 1.48–5.31; *P*=0.002) in the during-DCS compared with the post-DCS group. The other significantly associated parameters were sex (males), age (≥70), JCS (2–3 digits), and a history of heart failure or respiratory disease, with an AUC of 0.761. Details of the dental interventions are shown in Table 3. The number of patients who required dental intervention was considerably higher in the post-DCS group than in the pre-DCS group (*P*=0.002). The number of days between admission and dental intervention in the post-DCS group was significantly shorter than in the pre- and during-DCS groups (pre-DCS vs. post-DCS, *P* < 0.01; during-DCS vs. post-DCS, *P* < 0.0001). The dental interventions did not differ significantly between the three groups in terms of content; hygiene management was the main content, followed by hygiene guidance, denture adjustment, mucositis treatment, and tooth extraction. The procedures performed in less than 10% of the dental intervention patients (% of dental intervention patients) were as follows: hemostasis in 13 patients (5.4%); caries treatment, except for residual roots in seven patients (2.9%); and manual restoration for temporomandibular joint dislocation in two patients (0.8%). The few oral diseases observed in the patients at first visit were oral candidiasis (six patients; 2.5%), peri-implantitis (two patients; 0.8%), and hematoma (two patients; 0.8%). Difficulty in opening the mouth was observed in 17 patients (7.1%), and seven patients (2.9%) complained of a toothache. Twelve (5%) patients developed pneumonia after the dental intervention; 47 (67.1%) of the 70 patients who had denture adjustments were able to wear them at discharge. No sudden, adverse changes were observed during all the dental treatments.

## 4. Discussion

The assignment of a full-time dentist, who worked exclusively for the ward in collaboration with the nurses, reduced the incidence of nosocomial pneumonia in patients with acute stroke. In our previous survey limited to acute stroke patients referred to the rehabilitation department, we reported a decrease in pneumonia due to the introduction of DCS and the intervention of a speech-language pathologist [14], thus confirming that the DCS was effective in all stroke patients.

A comparison of the patient characteristics between the current study and those of a multicenter study comprising 176,753 stroke patients in Japanese hospitals [15] showed that the mean age (72 vs. 73), percentage of patients with cerebral infarction/hemorrhage/subarachnoid hemorrhage (57/31/12% vs. 69/23/8%) and the proportion of 0/1-/2-/3- (digit) JCS scores at admission (33/36/15/17% vs. 41/35/12/12%) were generally similar. Therefore, the patients included in this study might be considered typical of stroke patients in Japanese hospitals. Consistent with the results of previous studies [16–19], significant differences in the incidence of pneumonia among patients aged ≥70 years and those with decreased consciousness levels, heart failure, and respiratory diseases were observed in the current study. The incidence of stroke-associated pneumonia can vary, depending on the definition of pneumonia, the follow-up period, and the location of the study. A review study [20] reported a pneumonia incidence of 3.9%–23.8% (in mixed settings, including the acute phase). Thus, an incidence rate of 4.8% of the post-DCS group in this study may be less than that reported in previous studies.

Another factor that might have contributed to the decrease in the incidence of pneumonia in our study was the high number of patients who received swallowing rehabilitation by a speech-language pathologist during the early stages of hospitalization. Previous studies have reported that early onset swallowing therapy by a speech-language pathologist is effective in preventing pneumonia [21, 22]. In short, the synergistic effect resulting from early oral management by a dentist and swallowing rehabilitation by a speech-language pathologist might have proved beneficial for the patients.

Aoki et al. reported a reduction in the incidence of pneumonia following treatment by a multidisciplinary team for acute stroke patients involving dentists and dental hygienists [13]. In this study, the rates of patients who received oral care from dental professionals before and after intervention were 13% and 52%, respectively. A similar increase from 20% to 31% was also observed after the DCS in the current study. Alternatively, Brady et al. [23] examined the effect of oral healthcare [24] in stroke units located in four hospitals and stated that oral healthcare was not associated with the development of pneumonia. However, dental intervention in their studies was an out-of-hospital service, and timely intervention for acute oral problems might not be possible. In addition, there might be a lack of information sharing between the doctors and nurses in the hospital and the dental staff who work outside the hospital. Due to restrictions in the insurance system in most countries, hospitalized patients might not be able to receive dental services immediately during the acute phase, but in Japan, dentists can work exclusively in the wards of hospitals.

The time between the introduction of DCS and the onset of pneumonia control will be discussed. No significant difference in the incidence of pneumonia was observed between the pre- and during-DCS groups. This might be because a certain period is necessary for establishing the DCS. In an RCT involving dentists in oral care education, Ames et al. [25] reported an improvement in the oral assessment scores after a 1-year and 2-month educational intervention for nurses in three emergency units. The authors developed oral care procedures, educational videos, and booklets similar to those used in this present study. Malik et al. [26] conducted a web-based health education program on oral hygiene care for nurses in 10 hospitals that provided rehabilitation services. Improvements in oral healthcare attitudes and knowledge were observed in the intervention group after 1 and 6 months, respectively. One study reported an improvement in oral health following an 18-month dental intervention for acute stroke patients along with the provision of oral care instructions to the nurses [27]. These studies indicate that it takes some time to establish the skills and knowledge regarding oral care among the nurses; in the current study, a reduction in the incidence of pneumonia was observed about 1 year after the introduction of the DCS. The increase in the number of post-DCS dental interventions and the shortening of the period from hospitalization to the provision of the dental intervention may indicate the increase in the knowledge about the DCS among the nurses. In this study, only 2 hours of lecture and practical training were provided to the nurses during DCS, but oral hygiene instructions were provided to 12% (116/945) of all stroke patients in the ward. Thus, feedback to nurses about oral hygiene management in the ward may have improved nurses' oral health awareness and skills. Moreover, the committees involved in oral care might have compensated for the lack of lectures and practice time.

The oral assessment tool used in this study was developed independently; hence, its reliability and validity are yet to be examined. For the oral assessment, we did not adopt the Revised Oral Assessment Guide [28] because it requires a dental mirror for drying, and the items on voice and swallowing are based on the assumption that the patient can communicate. In addition, we did not introduce the the Oral Health Assessment Tool [29] because it is difficult for the nurses to evaluate the dental caries status and dentures; moreover, it is difficult to assess dental pain during the acute stroke phase. The process of oral assessment in the DCS does not require any special instruments other than a penlight, and the items can be assessed even without any prior knowledge about dentistry. By simplifying the evaluation, we were able to rapidly perform the triage. According to a recent study, the current guidelines on oral care are based on weak evidence and are not comprehensive [30]. The reasons for this include the lack of skills, knowledge, and attitudes on the part of the caregivers and the severity of the oral symptoms. The symptoms that interfere with oral care include mucositis, bleeding, mobility teeth, dislocation of the temporomandibular joint, and difficulty in opening the mouth. Dental treatment may reduce these symptoms and make it easier for the nurses to provide oral care.

In a previous study, 50–60-year-old stroke patients had more dental caries and periodontal disease than those without stroke [31]. In this study, it was impossible to examine using X-ray both caries and periodontitis because the patients were treated in a restricted hospital room and their general condition was unstable. However, about 10% of the patients who received interventions underwent extractions of mobile teeth, indicating the presence of severe periodontal disease. In addition, 36% of the dental intervention patients had severe dysphagia with salivary aspiration. For the oral care of patients with severe dysphagia, it is necessary to consider the handling of aspiration-conscious oral care products, including fluid volume and oral suction, and we believe that our study demonstrated such dental expertise. Furthermore, it has been pointed out that poorly fitting dentures may reduce nutritional intake [32], and the risk of a denture entering the pharynx is high [33]. In this study, about 30% of the dental intervention patients presented with denture incompatibility; denture treatment may have prevented hyponutrition, denture aspiration, or accidental ingestion.

One of the limitations of this study is that it was a retrospective, single-center, cohort study. Information about the pneumococcal vaccination, stroke severity other than the level of consciousness, causative organisms of pneumonia, smoking history, medications, and nutritional items other than BMI, which are associated with the development of pneumonia, could not be investigated. Furthermore, the effect of dental treatment is unknown because the timing, frequency, and content of interventions vary. Thus, additional studies are required to demonstrate the effects of dental treatment on stroke patients in the future.

## 5. Conclusion

A DCS consisting of lectures and practical training sessions, oral assessments, standardized oral care techniques, and information about the procedures for nurse-requested dental interventions was created by a full-time dentist dedicated to the ward at our hospital. As a result, the incidence of pneumonia in acute stroke patients decreased, thus indicating that a full-time dentist dedicated to a hospital ward consisting of stroke patients might aid in improving their condition.

## Figures and Tables

**Table 1:** Summary of the patients in the three groups.

		Overall *N* (%)	DCS			*P*-value
			Pre: April 1, 2012 to March 31, 2013; *N* (%)	During: April 1, 2013 to March 31, 2014; *N* (%)	Post: April 1, 2014 to March 31, 2015; *N* (%)	
Stroke		945	305	327	313	
Classification of stroke	Cerebral infarction	537 (56.8)	172 (56.4)	194 (59.3)	171 (54.6)	0.473
	Cerebral hemorrhage	295 (31.2)	91 (29.8)	96 (29.4)	108 (34.5)	
	Subarachnoid hemorrhage	113 (12)	42 (13.8)	37 (11.3)	34 (10.9)	
Admission by ambulance		679 (71.9)	233 (76.4)	233 (71.3)	213 (68.1)	0.067
Hospitalization route	Home	869 (92)	283 (92.8)	307 (93.9)	279 (89.1)	0.250
Sex	Male	512 (54.2)	153 (50.2)	188 (57.5)	171 (54.6)	0.178
Age	60>	144 (15.2)	56 (18.4)	55 (16.8)	33 (10.5)	0.120
	60–69	240 (25.4)	75 (24.6)	90 (27.5)	75 (24)	
	70–79	271 (28.7)	84 (27.5)	89 (27.2)	98 (31.3)	
	80–89	241 (25.5)	83 (23.9)	77 (23.6)	81 (25.9)	
	≥90	59 (6.2)	17 (5.6)	16 (4.9)	26 (8.3)	
BMI < 18.5 kg/m^2^		86 (9.1)	30 (9.8)	29 (8.9)	27 (8.6)	0.858
JCS	0 (alert consciousness)	312 (33)	90 (29.5)	116 (35.5)	106 (33.9)	0.397
	1-digit (is awake without any stimulus)	336 (35.6)	112 (36.7)	112 (34.3)	112 (35.8)	
	2-digit (can be aroused)	138 (14.6)	55 (18)	44 (13.5)	39 (12.5)	
	3-digit (cannot be aroused by any forceful stimuli)	159 (16.8)	48 (15.7)	55 (16.8)	56 (17.9)	
Comorbidities	Previous stroke	202 (21.4)	61 (20)	62 (19)	79 (25.2)	0.119
	Dementia	75 (7.9)	23 (7.5)	22 (6.7)	30 (9.6)	0.390
	Heart failure	72 (7.6)	26 (8.5)	25 (7.7)	21 (6.7)	0.696
	Renal failure	62 (6.6)	17 (5.6)	26 (8)	19 (6.1)	0.441
	Hypertension	735 (77.8)	231 (75.7)	256 (78.3)	248(79.2)	0.558
	Respiratory illness	103 (10.9)	33 (10.8)	34 (10.4)	36 (11.5)	0.903
	Hyperlipidemia	270(28.6)	74 (24.3)	105 (32.1)	91 (29.1)	0.090
	Diabetes mellitus	283 (29.9)	76 (24.9)	108 (33)	99 (31.6)	0.062
	Liver disease	65 (6.9)	15 (5)	20 (6.1)	30 (9.6)	0.058
Intervention within 3 days of hospitalization	Surgery	131 (13.9)	52 (17.1)	45 (13.8)	34 (10.9)	0.084
	Speech-language pathologist	776 (82.1)	256 (83.9)	266 (81.4)	254 (81.2)	0.601
	Dentistry	52 (5.5)	11 (3.6)	7 (2.1)	34 (10.9)	<0.0001^∗^

BMI, body mass index; JCS, Japan Coma Scale; DCS, dental care system. ^*∗*^*P* < 0.001 chi-square test, Pre-DCS versus Post-DCS, During-DCS versus Post-DCS. All comparisons used Pearson's chi-square test, and multiple comparisons used Bonferroni's adjustment.

**Table 2:** Relationship between pneumonia and each variable (logistic regression analysis).

		Odds ratio (95% CI)	*P* value
Male		1.81 (1.11–2.93)	0.016
Age ≥70 years (reference ≤69 years)		2.57 (1.48–4.43)	0.001
BMI <18.5 (reference ≥18.5)		1.23 (0.62–2.44)	0.556
JCS at ≥2 digits (reference ≤1 digit)		2.93 (1.82–4.81)	<0.0001
Heart failure		2.11 (1.06–4.23)	0.034
Respiratory illness		2.11 (1.17–3.80)	0.014
Nonintervention by a speech-language pathologist within 3 days of hospitalization		0.75 (0.41–1.38)	0.358
DCS
(reference Post)	Pre	3.16 (1.65–6.05)	0.001
	During	2.79 (1.48–5.31)	0.002
(reference During)			
	Pre	1.13 (0.68–1.88)	0.638

BMI, body mass index; JCS, Japan Coma Scale; DCS, dental care system.

**Table 3:** Dental intervention patients.

		Overall *N* (%)	DCS			*P*-value
			Pre: April 1, 2012 to March 31, 2013; *N* (%)	During: April 1, 2013 to March 31, 2014; *N* (%)	Post: April 1, 2014 to March 31, 2015; *N* (%)	
Number of patients (% of all stroke patients)		239 (25.3)	61 (20)	80 (24.5)	98 (31.3)	0.005^*∗*^
Number of dental visits ± SD (times)		5.8 ± 4.8	4.7 ± 4.6	6.3 ± 4.8	5.9 ± 4.9	0.055
Time from hospital admission to intervention ± SD (days)		12.2 ± 13.4	12.0 ± 9.5	15.6 ± 17.4	9.5 ± 10.9	<0.0001^*∗∗*^
Contents of dental treatment	Hygiene management	233 (97.5)	60 (98.4)	78 (97.5)	95 (96.9)	0.856
	Hygiene instruction	116 (48.5)	32 (52.5)	32 (40)	52 (53.1)	0.173
	Denture adjustment	70 (29.3)	15 (24.6)	17 (21.3)	38 (38.8)	0.025^#^
	Mucositis treatment	34 (14.2)	4 (6.6)	12 (15)	18 (18.4)	0.113
	Tooth extraction	24 (10)	3 (4.9)	6 (7.5)	15 (15.3)	0.069

*Initial examination*						
Number of remaining teeth	0	56 (23.4)	17 (27.9)	14 (17.5)	25 (25.5)	0.286
	1–9	55 (23)	16 (26.2)	18 (22.5)	21 (21.4)	
	10–19	63 (26.4)	11 (18)	21 (26.3)	31 (31.6)	
	20≤	65 (27.2)	17 (27.9)	27 (33.8)	21 (21.4)	
Eichner classification	A	35 (14.6)	8 (13.1)	16 (20)	11 (11.2)	0.552
	B	89 (37.2)	22 (36.1)	29 (39)	38 (38.8)	
	C	115 (48.1)	31 (50.8)	35 (43.8)	49 (50)	
Denture ownership	Full dentures	36 (15.1)	7 (11.5)	9 (11.3)	20 (20.4)	0.156
	Full and partial dentures	17 (7.1)	4 (6.6)	5 (6.3)	8 (8.2)	0.868
	Partial denture	19 (8)	4 (6.6)	5 (6.3)	10 (10.2)	0.56
Crusting on the lips		33 (13.8)	5 (8.2)	11 (13.8)	17 (17.3)	0.266
Oral dryness		123 (51.5)	29 (47.5)	48 (60)	46 (46.9)	0.172
Dysphagia (saliva aspiration)		85 (35.6)	17 (27.9)	31 (38.8)	37 (37.8)	0.344

DCS, dental care system. ^*∗*^*P*=0.002, Pre-DCS versus Post-DCS. ^*∗∗*^*P* < 0.01, Pre-DCS versus Post-DCS, *P* < 0.0001, During-DCS versus Post-DCS. ^#^*P*=0.012, During-DCS versus Post-DCS. The number of dental visits and the time from admission to intervention were calculated using Wilcoxon signed-rank sum test. Everything else is Pearson's chi-square test. Multiple comparisons are Bonferroni corrections.

## Data Availability

The data used to support the findings of this study are available from the corresponding author upon request.

## Supplementary Materials

Supplementary Materials Oral Assessment Sheet.
