# Supplementary material for: Oral Management by a Full-Time Resident Dentist in the Hospital Ward Reduces the Incidence of Pneumonia in Patients with Acute Stroke
Source: Int J Dent. 2022 Jul 22;2022:6193818. doi: 10.1155/2022/6193818 (PMC9337935; doi:10.1155/2022/6193818)
Supplement: Supplementary Materials — Oral Assessment Sheet. [file 6193818.f1.pdf]

Oral Assessment Sheet

ID.________________

Name.__________________

Denture (maxillary): No・Yes, (mandibular): No・Yes    Oral intake: No・Yes → In the case of Yes（Denture incompatibility: No・Yes）

About Assessment

(1) Phlegm (Yes) → dry or viscous phlegm is also evaluated

(2) Dryness (Yes) → Almost no saliva

(3) Food residue (Yes) → 5 mm or more

(4) Lichen on tongue (Yes) → More than 2/3 of the dorsum of tongue

(5) Difficulty in opening mouth (Yes) → 2 side fingers or less

(6) Ulcers (Yes), (7) Bleeding (Yes) → Check in the following order: lip and cheek mucosa, gingiva, palate, tongue, and floor of the mouth

| Month/day | ／ | ／ | ／ | ／ | ／ | ／ | ／ |
|---|---|---|---|---|---|---|---|
| (1) Phlegm | No ・ Yes | No ・ Yes | No ・ Yes | No ・ Yes | No ・ Yes | No ・ Yes | No ・ Yes |
| | dry or viscous | dry or viscous | dry or viscous | dry or viscous | dry or viscous | dry or viscous | dry or viscous |
| (2) Dryness | No ・ Yes | No ・ Yes | No ・ Yes | No ・ Yes | No ・ Yes | No ・ Yes | No ・ Yes |
| (3) Food residue | No ・ Yes | No ・ Yes | No ・ Yes | No ・ Yes | No ・ Yes | No ・ Yes | No ・ Yes |
| (4) Tongue coating | No ・ Yes | No ・ Yes | No ・ Yes | No ・ Yes | No ・ Yes | No ・ Yes | No ・ Yes |
| (5) Difficulty in opening mouth | No ・ Yes | No ・ Yes | No ・ Yes | No ・ Yes | No ・ Yes | No ・ Yes | No ・ Yes |
| (6) Ulcers | No ・ Yes | No ・ Yes | No ・ Yes | No ・ Yes | No ・ Yes | No ・ Yes | No ・ Yes |
| (7) Bleeding | No ・ Yes | No ・ Yes | No ・ Yes | No ・ Yes | No ・ Yes | No ・ Yes | No ・ Yes |
| Total points | | | | | | | |
| (No; 0 points , Yes; 1 point) | | | | | | | |
| Care equipment used | □ Sponge brush | □ Sponge brush | □ Sponge brush | □ Sponge brush | □ Sponge brush | □ Sponge brush | □ Sponge brush |
| | □ Toothbrush | □ Toothbrush | □ Toothbrush | □ Toothbrush | □ Toothbrush | □ Toothbrush | □ Toothbrush |
| | □ Tongue brush | □ Tongue brush | □ Tongue brush | □ Tongue brush | □ Tongue brush | □ Tongue brush | □ Tongue brush |
| | □ Interdental brush | □ Interdental brush | □ Interdental brush | □ Interdental brush | □ Interdental brush | □ Interdental brush | □ Interdental brush |
| | □ Moisturizer | □ Moisturizer | □ Moisturizer | □ Moisturizer | □ Moisturizer | □ Moisturizer | □ Moisturizer |
| | □ Vaseline | □ Vaseline | □ Vaseline | □ Vaseline | □ Vaseline | □ Vaseline | □ Vaseline |
| | | | | | | | |
| | | | | | | | |
| Free Comment | | | | | | | |
| Pattern* | A, B, C, D | A, B, C, D | A, B, C, D | A, B, C, D | A, B, C, D | A, B, C, D | A, B, C, D |
| Nurse sign / care time | / | / | / | / | / | / | / |
| Dental hygienist sign / care time | / | / | / | / | / | / | / |

*Pattern classification of oral contamination

| | Phlegm (+), Pharyngeal suction (+) | | Phlegm (-), Pharyngeal suction (-) | |
|---|---|---|---|---|
| Oral mucosa | dryness | viscosity | dryness | normal |
| Pattern | A | B | C | D |
| Number of oral care per day for parenteral patients | 2-3 times/day | 2 times/day | 1-2 times/day | 1-2 times/day |

Supplementary Table 1. Oral Assessment Sheet
